# Unstable crop yields reveal opportunities for site-specific adaptations to climate variability

**DOI:** 10.1038/s41598-020-59494-2

**Published:** 2020-02-19

**Authors:** Rafael A. Martinez-Feria, Bruno Basso

**Affiliations:** 1 Department of Earth and Environmental Sciences, Michigan State University, East Lansing, MI 48824 USA; 2 W.K. Kellogg Biological Station, Michigan State University, Hickory Corner, MI 49060 USA

**Keywords:** Hydrology, Agroecology

## Abstract

Water deficit and water excess constitute severe stresses that limit crop yield and are likely to intensify as climate becomes more variable. Regional crop production aggregates for the US Midwest indicate widespread yield losses in past decades due to both extreme rainfall and water limited conditions, though the degree to which these weather impacts are related to site-specific factors such as landscape position and soils has not been examined in a systematic manner. This study offers observational evidence from a large sample of commercial crop fields to support the hypothesis that landscape position is the primary mediator of crop yield responses to weather within unstable field zones (i.e., zones where yields tend to fluctuate between high and low, depending on the year). Results indicate that yield losses in unstable zones driven by water excess and deficits occur throughout a wide range of seasonal rainfall, even simultaneously under normal weather. Field areas prone to water stress are shown to lag as much as 23–33% below the field average during drought years and 26–33% during deluge years. By combining large-scale spatial datasets, we identify 2.65 million hectares of water-stress prone cropland, and estimate an aggregated economic loss impact of $536M USD yr^−1^, 4.0 million tons yr^−1^ of less CO_2_ fixed in crop biomass, and 52.6 Gg yr^−1^ of more reactive N in the environment. Yield stability maps can be used to spatially implement adaptation practices to mitigate weather-induced stresses in the most vulnerable cropland.

## Introduction

The United States Midwest supplies about a third of the world’s corn (*Zea mays*) and soybean (*Glycine max* L [Merr.])^1^, but more frequent weather extremes—including excessive rainfall and drought conditions—increasingly threaten crop yields^2,3^. Indeed, analyses of crop production aggregates for the region have indicated substantial yield losses not only during drought, but also in years with excess rainfall^4–6^. Both water deficits and water excess constitute severe stresses that limit crop growth and yield. Plant physiological mechanisms for conserving water and maintaining turgor during drought inhibit photosynthesis and leaf area expansion, while extreme heat that can develop under drought conditions results in wasteful respiration, accelerates phenological development and can cause irreversible damage to plant tissues^7–9^. Waterlogging following the occurrence of heavy rains decreases seedling emergence, suppresses root growth as a consequence of oxygen deficit and reduces crop nutrient uptake^10–12^. These crop stresses can be expected to intensify as climate becomes more variable.

Regional-level assessments are instrumental to understanding the magnitude and extent of climate-driven impacts on crop productivity, but they cannot capture important field-to-field or sub-field variability. In fact, recent high-resolution crop yield estimates derived from satellite imagery^13^ have indicated that about 28% of all corn and soybean cropland in the Midwest is temporally unstable, meaning that yields in these zones tend to fluctuate between high and low, depending on the year. These shifts between favorable and unfavorable growing conditions in unstable subfield zones could be explained by patterns of overland water flow and accumulation^14,15^. While soil physical properties regulate water infiltration and soil water holding capacity, position in the landscape influences the amount of water that is lost through runoff and gained through run-on, creating areas that are more inclined to become water limited or waterlogged^11^. This dynamic has been widely documented in field experiments, where areas with higher relative elevation or slope generally yield less in normal or dry years^15–19^, whereas areas that collect overland runoff show suppressed yields during wet years^15,18,20,21^.

**Figure 1:**
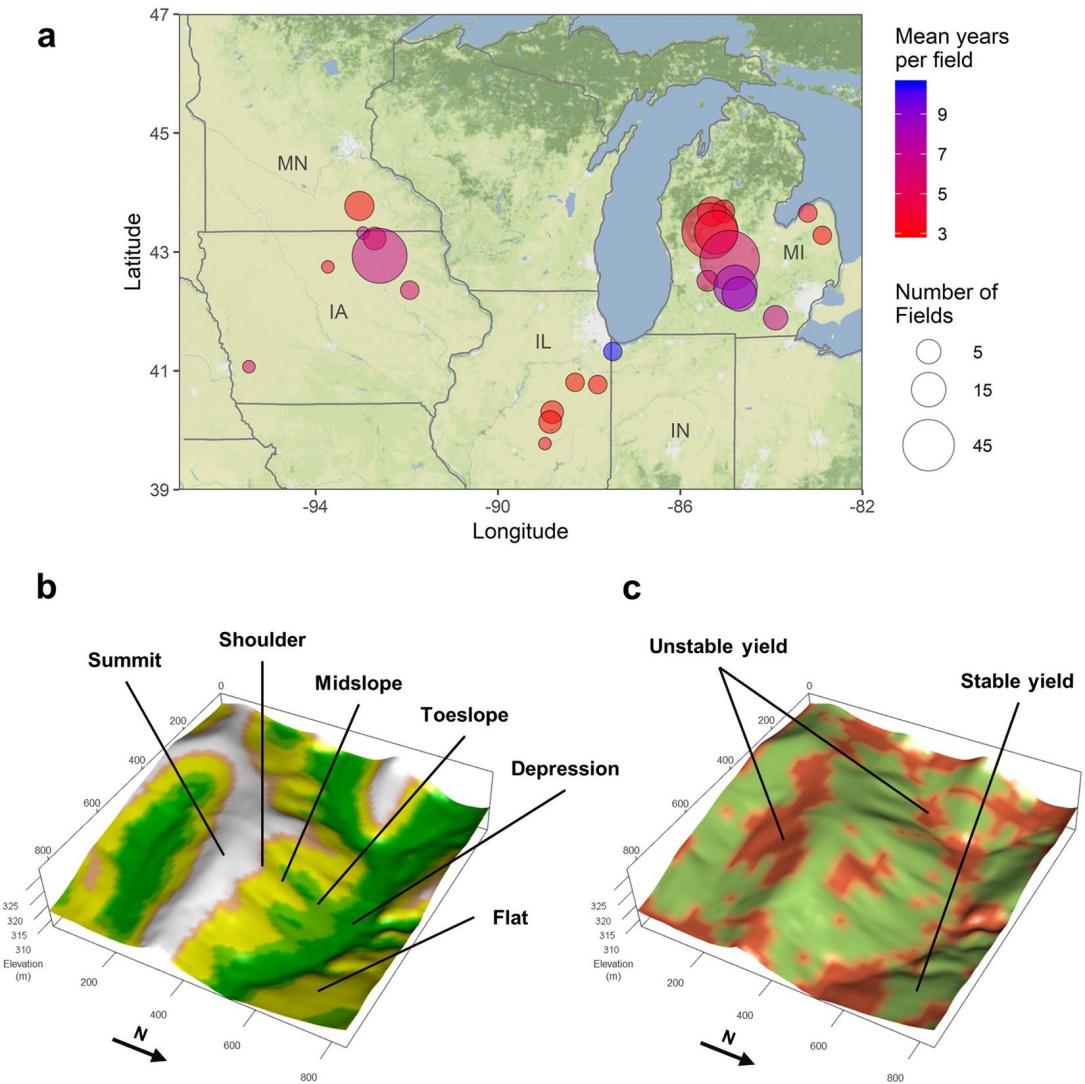
Location of sample crop fields and subfield classifications. (**a**) Geographic distribution of sample crop fields used for the analysis (see Table S1 for details). Each field was subdivided into 0.09 ha grid cells, which were then classified according to: (**b**) position in the landscape based on digital elevation models; and (**c**) long-term crop yield stability derived from historical yield maps collected with combine yield monitors (at least 3 yr. for each field). Color ramp in (**b**) indicates topographic position index (TPI) and in (**c**) areas of stable and unstable yields.

While the weather-landscape position interaction is widely recognized as a major driver of crop yield spatio-temporal variability^14,22,23^, its importance relative to other sources of variation such as soil type or previous yield history remains unclear. In addition, the extent and magnitude of the associated yield gaps have not been examined in a systematic manner. Therefore, here we use observational data from a large sample of commercial crop fields (Fig. 1a) to examine the hypothesis that landscape position is the primary mediator of crop yield responses to weather within unstable field zones, leading to contrasting responses within the same field. We then combine large-scale spatial datasets with subfield designations of yield stability classes and landscape positions to identify and quantify cropland that is most vulnerable to hydric stresses (i.e., too little or too much water) and estimate yield gaps and scale up impacts. Our aim is to provide a baseline for the potential improvements in yield and resilience that may be achieved by site-specific implementation of management or genetic adaptations.

## Results

### Landscape position as mediator of crop yield responses to weather in unstable zones

To test the hypothesis that landscape position is the primary mediator of crop yield responses to weather within unstable field zones, we examine a set of 305 corn and soybean fields in five Midwestern states (Fig. 1a; Table S1), segmented according to site-specific yield stability and landscape position classifications (Fig. 1b,c). These sample fields encompass a wide heterogeneity of landscape positions (Fig. S1). Unstable zones across all fields represented 27% of all grid cells, which is remarkably similar to the satellite-derived estimate of 28% for the entire Midwest^13^.

**Figure 2:**
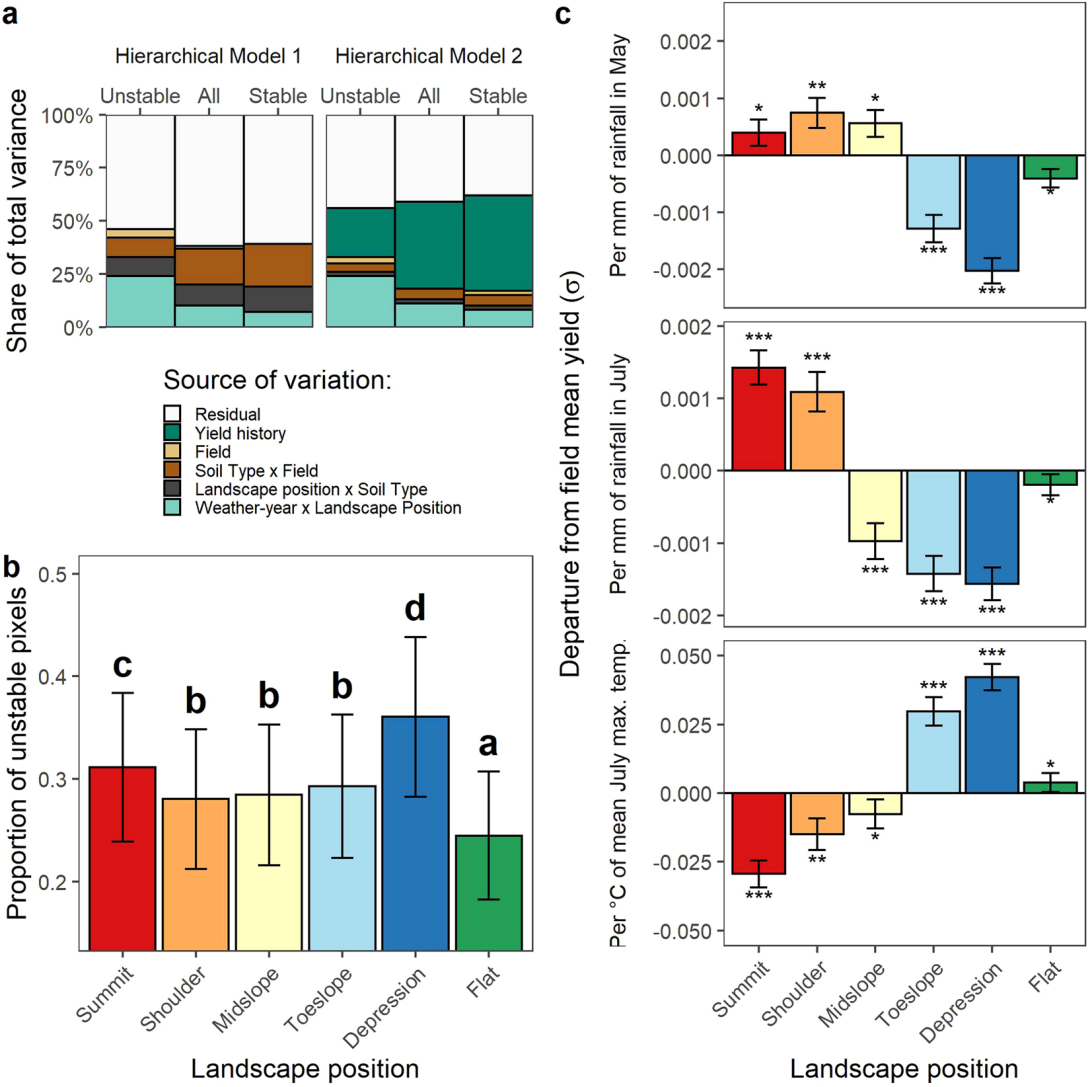
Variance components and crop-weather responses in unstable zones across landscape positions. (**a**) Variance components (expressed as percent of total variance) for hierarchical linear models describing variability in temporal yield responses within crop fields. (**b**) Proportion of unstable yield map pixels, that is with a large degree of temporal variability. Landscape positions with the same letter indicate that they are not significantly different (Tukey adjustment, p < 0.05). (**c**) Standardized yield response to May rainfall, July rainfall and July average maximum temperature across landscape positions. Significance codes: *p < 0.05, **p < 0.01; ***p < 0.001. Error bars indicate standard error of the estimate.

We identify four sources of variation in our dataset that may explain spatial patterns of temporal crop yield responses: field, soil type, landscape position and weather-year. Variance decomposition with hierarchical linear models indicate that the greatest share of the non-error variance in stable zones can be attributed to soil type within fields whereas other sources of variation play minor roles (Fig. 2a). In unstable zones, soil type within fields accounts for only 10% of the total variance, while the interaction of weather-year within landscape position becomes much more important. Yield history (i.e., whether the grid cell-wise mean is above or below the field mean^22^) as explanatory variable captures the variance associated with space (i.e., field, soil type and landscape position), but not spatiotemporal responses (i.e., weather-year and landscape position interactions; Fig. 2a). The decrease in the usefulness of yield history as explanatory variable in unstable zones is clearly related to variance captured by the interaction of weather-year and landscape position. When variance components are calculated for each field independently, we find that those fields where soil explains little of the variance are in those where the weather-year and landscape position interactions hold the greatest explanatory power (Fig. S2).

Further analysis shows that crop yield stability differs significantly among landscape positions (p < 0.001; Table S2). In the sample fields examined, the proportion of unstable grid cells (i.e., high temporal variation) are greatest in the depression and summit positions, followed by shoulder, midslope and toeslope positions, and then flat cropland (Fig. 2b). The same general pattern was observed when the data was analyzed by region (Fig. S3). This provides evidence that unstable zones are also common in higher elevation areas and not just in depressional areas^14^.

In unstable zones, crop plants exhibit high yields in dry years and low yields in wet years if located in depressional areas, whereas the opposite is true for summit and sloping areas. This contrasting response becomes evident when examining site-specific yield responses to weather during critical periods of crop growth (i.e., May and July). In the Midwest, the month of May represents the period of corn and soybean planting and seedling emergence, and July encompasses the period around flowering. Greater rainfall in May and July is associated with increases in standardized yield per unit of mm in the summit and shoulder positions, although the response is much greater for July rainfall (Fig. 2c; Table S3). In contrast, increases in rainfall both in May and July are associated with large negative departures from the field mean yield in the toeslope and depression positions. On the other hand, higher daily temperatures in July are related to a positive standardized yield response in the lowland portions relative to the field mean, and to negative responses in the upland positions. Not surprisingly, crop plants in the flat areas are the most resilient, showing to be the least sensitive to weather variability (Fig. 2c). The same general pattern is observed when the data are analyzed by region and crop (Figs. S4 and S5).

In addition, we examine aerial thermal imagery (2 m resolution) of crop canopies collected during the month of July for 60 of the sample fields (Fig. S6a). These data indicate significantly warmer crop canopy temperatures in July in the upland positions, whereas flat and lowland positions tend to have cooler canopies (Fig. S6b; Table S4). The warmer canopy temperatures could suggest that upland positions show greater signs of stress related to water-deficits occurring during this critical period of crop yield determination^24^. It should be noted, however, that most of the fields with canopy temperature measurements in our dataset are located in Michigan (57 out of 60), which tend to have low quality, predominantly sandy soils (Figs. S7–S9). It is possible that in more productive fields (e.g., Iowa), depressions may also experience a high departure from mean canopy temperature, especially in years with wet May-June, and dry July-August.

### Yield gaps in water-stress prone landscape positions

**Figure 3:**
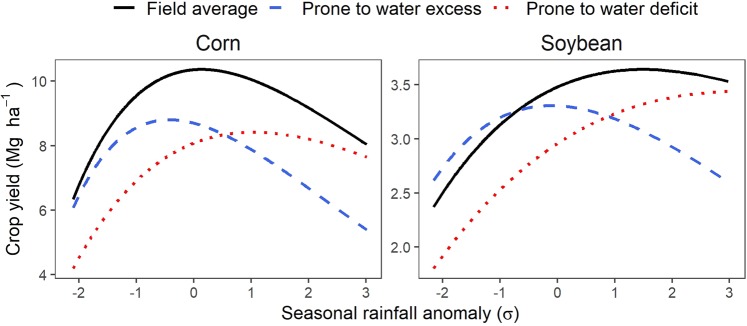
Average yield response to seasonal rainfall in landscape positions prone to water stress compared to the field average. Landscape positions prone to water excess are defined as unstable yield areas located in the toeslope and depressions, whereas landscape positions prone to water excess are unstable yield areas in the summit, shoulder and midslope. Response curves were calculated using data from the sample fields, by combining the non-linear response of field average yield to seasonal rainfall anomaly (solid black line) and OLS regression models for site-specific yields relative to the field average (see Methods and Figs. S8 and S9 for details).

Relative to the field average yield, the unstable areas in upland positions (summit, shoulder and midslope) seem to be the most prone to yield losses during dry years, whereas excess rainfall years are corelated to yield decline in unstable lowland positions (toeslope and depression; Fig. 3; Figs. S10 and S11). Yields in zones prone to water deficits can be as low as 23–33% below the field average in years of low rainfall (−2σ rainfall anomally; Fig. 3) but are comparable to the mean in extremely wet years (+3σ; Fig. 3). This is almost the reverse in zones prone to excess water, with yields 26–33% below the field average in wet years (+3σ).

### Extent of landscape-mediated impacts

**Figure 4:**
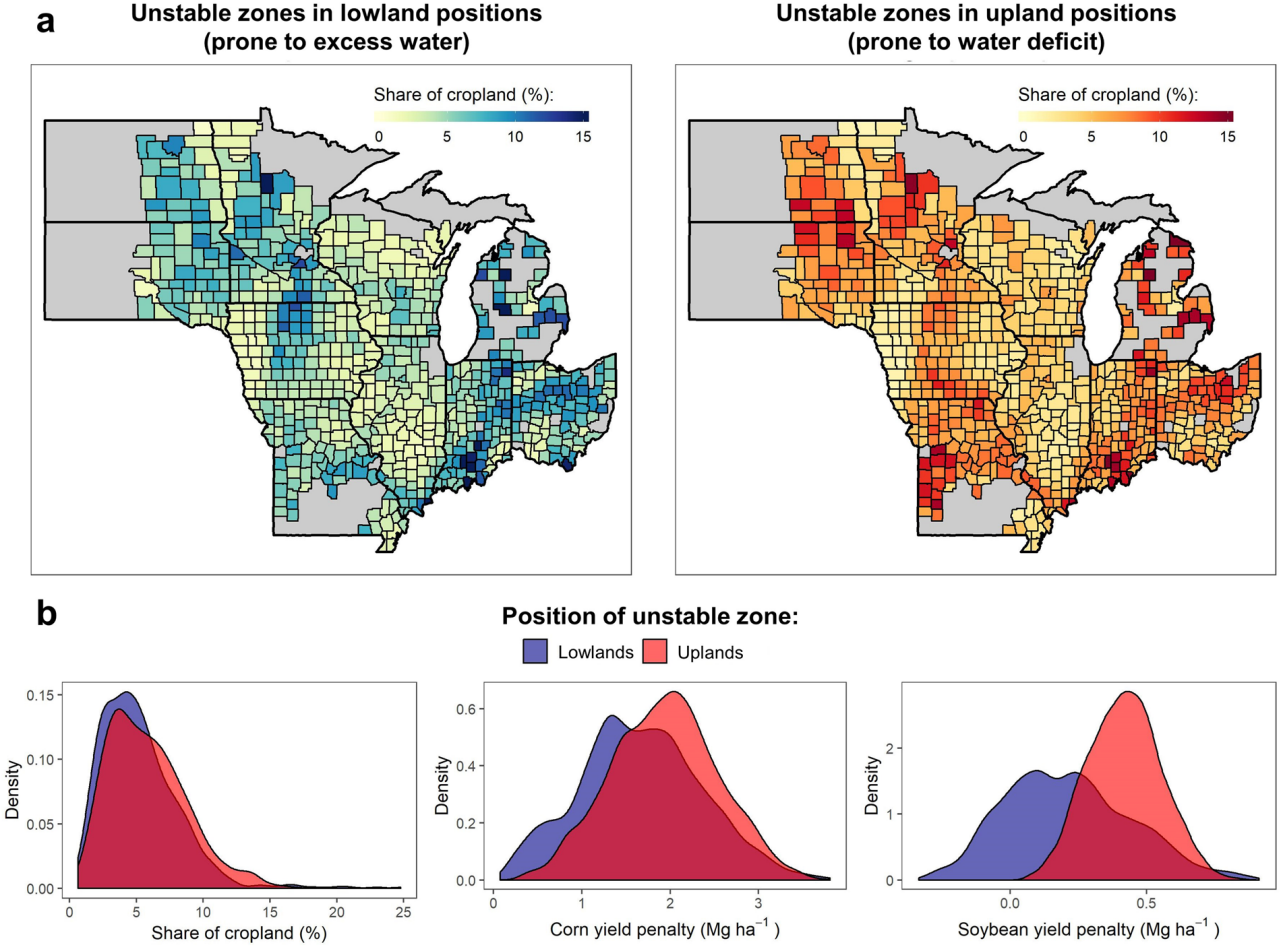
Geographic distribution and estimated yield gaps of unstable yield areas in the US Midwest. (**a**) Geographical distribution of subfield zones prone to water excess and deficits within corn and soybean fields in the US Midwest. Estimates are derived from a published spatial dataset of subfield yield stability classes^13^ and regional subfield landscape positions classifications (Fig. S7). (**b**) Distribution of estimated yield penalty in water stress-prone cropland compared to the county average for the period (2007–2016). These estimates are based on response curves developed using the measured yield map data (Fig. S5), county-level reports of corn and soybean yields (Figs. S11 and 12), and seasonal rainfall records (Fig. S13).

Using subfield designations of yield stability classes and landscape positions for Midwestern corn and soybean fields (Fig. S12), we estimate that in the Midwest there are roughly 2.7 million ha (~10% of total area) which are highly sensitive to excess water or water deficits, though these are clustered in a few areas in the northern and eastern Corn Belt (Fig. 4a). Assuming that our sample fields are broadly representative of Midwestern cropland (Fig. S11), we estimate that during 2007–2016, yield in water stress-prone cropland lagged on average 15% of county-level yields (Table 1; Fig. 4b). On the aggregate this amounted to 2.8 and 0.4 million tons yr^−1^ of lost corn and soybean grain production for the whole region, with an estimated economic impact of $536M USD yr^−1^.

**Table 1:** Estimated weather impacts on water stress-prone cropland between 2007 and 2016.

	Area (M ha-1)	Yield penalty^*^ (% of NASS county yield)	Less grain (M tons y^−1^)	Lost monetary value^†^ (M USD y^−1^)	More reactive N^†‡^ (Gg N y^−1^)	Less CO_2_ captured in crop residues^§^ (M tons CO_2_ y^−1^)
**Unstable cropland prone to excess water**						
Corn	0.73	17 (2.4–33)	1.3	182	24.4	1.6
Soybean	0.62	7 (0–27)	0.15	49	—	0.2
**Unstable cropland prone to water deficit**						
Corn	0.71	21 (4.6–36)	1.5	210	28.2	1.9
Soybean	0.59	14 (2.3–25)	0.27	95	—	0.3
**Total**	**2.65**	**15** (**7**–**19**)	**3.22**	**536**	**52.6**	**4.0**

^*^Values in parentheses represent ranges.

^†^Calculated assuming corn and soybean prices of 140 and 350 USD/ton, respectively.

^‡^Assuming corn nitrogen concentration is 1.2 and 0.8% in grain and stover, respectively, and 0.45 harvest index.

^§^Assuming plant biomass is 40% C and harvest index of 0.45, all on a dry-matter basis.

Based on the yield losses associated to site-specific responses to drought and deluge, the environmental impacts amount to roughly 4.0 million tons yr^−1^ of CO_2_ that were not fixed in crop biomass and returned to soils, and 52.6 Gg yr^−1^ of reactive N that was not taken up by crops and was likely lost to the environment (Table 1). Here these impacts are estimated relative to the field or county average yield, which is likely a conservative estimate as the actual water-limited yield potentials are probably higher, particularly in lowland areas.

## Discussion

Enhancing the resilience of agricultural production to weather variability and climate change requires a better understanding of the underlying processes that drive weather-induced crop stress and yield loss. While temperature and precipitation trends in the US Midwest have so far been beneficial for corn and soybean production (accounting for about a quarter of the yield-increase trend in recent decades^25–27^), regional yields are also simultaneously becoming more vulnerable to weather shocks^4–6,28^. The trend of intensifying rainfall in the Midwest^3,29^, in particular, poses a critical threat as extreme rainfall can also result in significant production losses^4,30^. In fact, a recent study that examined long-term (1981–2016) trends for corn production records and insurance data in the US^4^ indicated that excessive rainfall reduced yields up to 34%, which were comparable to those due to extreme drought (37%). Yet, yield losses due to excess water remain much less studied than drought stress factors^4,31^, which is a crucial gap and limits our ability to adapt crop production systems.

Critically, weather cannot be assumed to impact crop stand uniformly because, as demonstrated here, position in the landscape mediates the microenvironment that plants experience, which can substantially mitigate or accentuate weather-induced crop stress (Figs. 2c and 3; Fig. S6b). Indeed, the evidence provided by our large sample of Midwestern fields supported our hypothesis that the contrasting crop responses to weather across landscape positions are the primary drivers of yield variability in unstable zones (Fig. 2a), which represent more than a quarter of cropland in the Midwestern US^13^. The 2.7 million hectares of cropland identified as most prone to water stress are estimated to lag as much as 23–33% below the field average during drought years and 26–33% during deluge years compared to the field average yield on a given year, amounting to substantial environmental and economic cost (Table 1). This might suggest that a sizable portion of yield loss during years of extreme weather-induced stress might originate from these most vulnerable zones, and that stable, mostly flat cropland might be better able to withstand weather variability shocks (Figs. 2b,c and S10). Nevertheless, it is important to highlight that yields in unstable zones appear to be limited by water-stress even under nonextreme rainfall (~mean seasonal rainfall; Fig. 3), implying that situations where fields are simultaneously affected by excess water in certain areas and by water deficits in other areas, might be more common than what is generally assumed.

An important uncertainty in our analysis is the effect of artificial subsurface tile drainage systems on the yield stability and landscape-mediated yield responses, given that no information was available for our sample fields on which had tiles and their location within the field. Yet, subsurface drainage is a widespread practice in the Midwest^32^. It may be expected that fields in our dataset located in regions where tile drainage is more prevalent (e.g., Minnesota and Iowa) would tend to have greater stability in depressions than in regions with less tile drainage (e.g., Michigan), perhaps comparable to that of flat cropland. At the very least, we might expect yield responses to excessive rainfall in depressions to be less pronounced. The former does not seem to be the case (Fig. S3), whereas for the latter, it is unclear if the muted landscape-mediated yield responses in Minnesota and Iowa (Fig. S4) are due to tile drainage, or to the overall larger proportion of unstable pixels in those fields (Fig. S3). In fact, it has been previously shown that tile drainage is not a major factor when determining the regional variations in excessive rainfall impact^4^ because regions that tend to have poorly drained soils, and thus are more vulnerable to waterlogging, are also those that have greater proportion of land under artificial drainage. Therefore, grouping fields by region to evaluate the effect of tile drainage here should be interpreted with caution because of many confounding factors (e.g., soil hydrology, productivity level, etc.).

It should also be noted that not all unstable field zones are located in upland or lowland positions. In fact, nearly one third of unstable zones in our dataset occur in flat areas. This may indicate that factors such as intermittent disease, weeds or planting errors are also important to consider when establishing the causes for the lack of site-specific yield stability. Additionally, the influence of soil characteristics cannot be completely separated from position in the landscape. For example, long-term soil erosion and deposition dynamics can cause soils in the upland and sloping positions to be of lower soil organic matter and soil microbial function^33^, coarser texture and occasionally shallower depth (Figs. S7–S9). These factors substantially influence components of water budgets (e.g., runoff, depth to water tables, and drainage), nutrient budgets (e.g., mineralization, losses and gains) and the plant physical environment (e.g., rooting depth)^11,31^, all of which affect yield potential and variability. Thus, landscape position should be thought of as an integrative characteristic that encompasses complex interactions between water and nutrient cycling, allowing us to infer and predict responses to environmental variability. It is then that by coupling data on landscape position with spatial yield variability analysis (i.e., site-specific information of past yields), it is possible to identify areas where potential yields are high but are often suppressed by excessive or deficient water. In this sense, crops themselves are the best indicators of field areas that would most benefit from adaptations.

### A framework for adapting crop production landscapes

**Figure 5:**
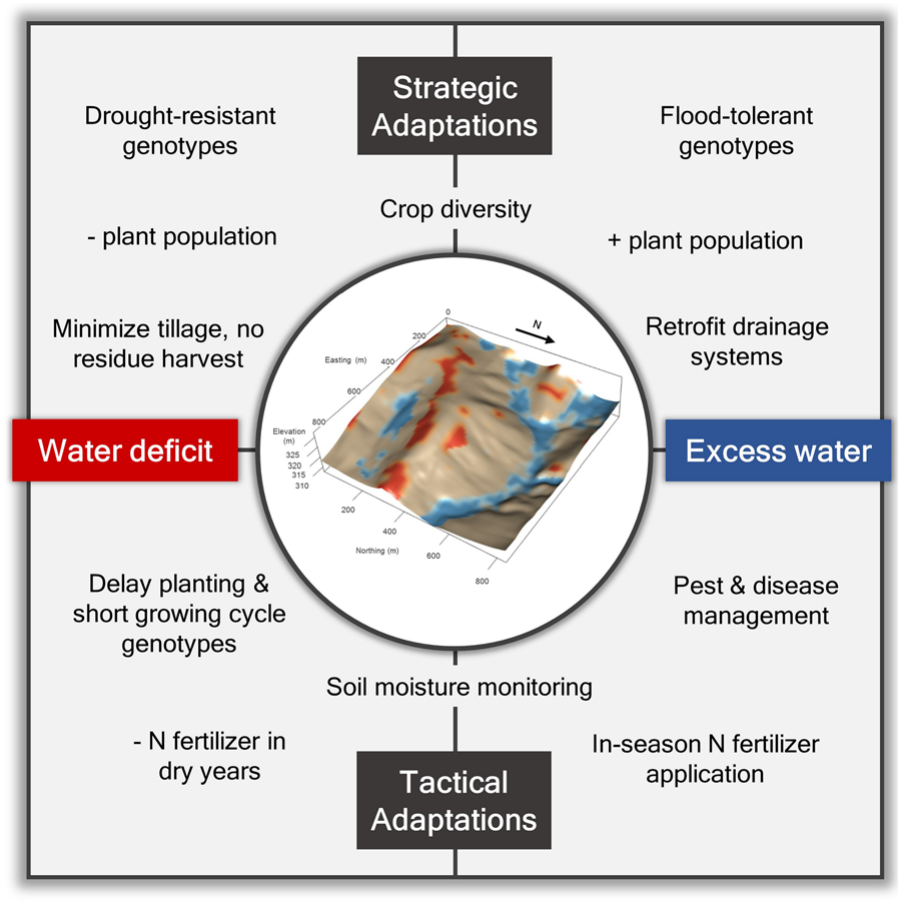
Summary of site-specific adaptations to increasing weather variability. Cropland prone to water deficit or excess can be identified as those subfield areas with a history of unstable yields which are located in relatively higher or lower positions within the field, respectively. Strategic adaptations refer to pre-season selection of management practices derived from long-term responses shown to reduce the risk of yield losses. Tactical adaptations are decisions made during a crop season to adjust management to the set of growing conditions being experienced^34^.

The expected increase in climate variability with future climate change can be used to estimate the risk probability of reduced yields and identify possible strategic and tactical adaptations^34^ for distinct landscape positions (some examples are summarized in Fig. 5). For instance, increasing plant densities in excess water-prone zones might help mitigate the negative effects of poor stand establishment during wet years^10^ while still supporting high yields under dry years. Drainage can also be improved in many of the poorly drained soils of the Midwest to increase yield and reduce waterlogging with its associated negative effects^32^ (e.g., effects on germination, denitrification, anoxia). This is crucial as the design of current drainage systems may not be adequate to accommodate increases in drainage requirements due to intensifying rainfall in the region^35^. On the other hand, drought-adaptation strategies that decrease the crop water demand during peak summer temperatures such as lower plant densities, later planting or faster-development (shorter duration growth period) varieties might help prevent water stress in upland areas^26^, but likely at the cost of lowering yield potential in these zones. Tactical management approaches such as in-season adjustment of N fertilizer inputs guided by weather forecasting, remote sensing and crop modeling^34,36^ can also help support higher yields in zones that are likely to benefit under favorable weather, and improve N-use efficiency in these zones under unfavorable weather.

The development of flood- and drought-tolerant genotypes also offers promising options for enhancing the resilience of crops to weather variability. Improvements in tolerance to short-term soil water saturation are tapping into known plant mechanisms to withstand root anoxic conditions such as aerenchyma formation, greater activity of the glycolytic pathway and fermentation enzymes, and antioxidant defense compounds^12^, while drought tolerance seeks to enhance stomatal regulation, osmotic adjustment, root architecture and plasticity^8,9,11^. However, crop traits for tolerance to flood and drought may not co-occur in the same genotype due to fitness tradeoffs^37^. Hence, coupling yield stability and landscape position information offers a spatial framework for experimentally testing novel germplasm in breeding programs or agronomic management, and ultimately for climate adaptation. In this sense, this proposed approach would allow matching genotypes or management adaptations to specific subfield microenvironments in which given weather-induced stresses are expected to be most pronounced or frequent. Therefore, integrated research is urgently needed to examine the feasibility of such strategies for climate-adaptation, and more broadly to determine its significance for crop production.

## Methods

### Sample fields and crop measurements

We used yield monitor datasets (i.e., yield maps) for 305 sample fields in Illinois, Indiana, Iowa, Michigan and Minnesota (Fig. 1a). The fields were cropped primarily with corn and soybeans, with a small number also including wheat in rotation. Field size ranged from 10 to 184 ha (34 ha on average), representing a total area of 10,544 ha (Table S1). Each unique field contained at least 3 years of end-of-season yields, collected within 2004–2017. Raw monitor yield data was adjusted to standard grain moisture (15.5% and 13% corn and soybean moisture, respectively) and cleaned as described in a previous study^14^.

Crop canopy thermal imagery during the month of July were collected at 60 of the fields (Fig. S6a) by a commercial provider (AirScout Inc., Monee, IL, USA) using the aircraft-mounted 9640P thermal sensor (Infrared Cameras Inc., Beaumont, TX, USA). Canopy temperature readings from the thermal band (7–14 µm) were reported as °C (range 12–46 °C) at a 2 m resolution. The georeferenced point-based yield and canopy data were transformed into raster format and resampled to 1-arc second (~30 m) resolution, so that it matched that of the digital elevation model (see below).

For further analysis, we standardized the absolute raster values of yield (Mg ha^−1^) and canopy temperature (°C) by subtracting the site-year wise mean value and dividing by the standard deviation. In these cases, the standardized values had a mean of zero and standard deviations (σ) as units.

### Landscape position classification

For each field, we obtained Digital Elevation Models (DEMs) at 1-arc second resolution (~30 m) from the National Elevation Dataset (NED)^38^. DEMs were used to calculate local topographic slope and topographic position index (TPI) for each pixel. Topographic slope was computed using the vector-based algorithm^39^ (i.e., 4 neighbors). Topographic position index (TPI) was calculated with the following equation:

1 $$TPI={Z}_{i}-{\bar{Z}}_{n}$$

where *Z*_*i*_ is the elevation for the *i*^th^ pixel, and ${\bar{Z}}_{n}$ is the mean elevation for the surrounding neighborhood. Here, we used a neighborhood of 9 × 9 pixels (~7.3 ha). The computed slope and TPI were used to classify each field pixel into five landscape positions (Fig. 1b): summit (TPI ≥ 1SD), shoulder (1SD > TPI ≥ 0.5SD), midslope (0.5SD > TPI ≥ −0.5SD & Slope ≥ 2%), toeslope (−0.5SD > TPI ≥ −1SD), depression (TPI < −1SD) and flat (0.5SD > TPI ≥ −0.5SD & Slope < 2%), where SD is the standard deviation of TPI values for our entire dataset (0.6 m).

Summit is the uppermost position on a hillslope. It represents a local interfluve, meaning that it receives little overland flow and contributes runoff to downslope positions. The shoulder position is a convex transitional zone at the base of the summit and above the midslope. The footslope lays at the base of hillslopes. Depressions are concave regions that receive most of overland upslope flow. Flat positions show very low local variation in elevation and are not located within sloping terrain.

### Temporal stability classification

We followed an established protocol for yield stability analysis^40^ to partition fields into stable and unstable zones (Fig. 1c). For every pixel of every field, we calculated the mean (µ) standard deviation (σ) of the standardized yields across all the years available for that field. Then each pixel was recoded as *stable* if σ < 0.75 and *unstable* if σ ≥ 0.75. Likewise, yield history of each pixel was deemed as *low-yielding* if µ < 0 and *high-yielding* if µ ≥ 0. Yield map pixels which had at least one missing value across the different years were excluded from the computation.

### Weather and soils

Historical daily weather observations (1988–2017) were extracted for each field from the Daymet dataset^41^, downscaled to a 1 × 1 km resolution. Daily rainfall and temperature were aggregated to monthly values. Local soil information was retrieved from the Soil Survey Geographic (SSURGO) database^42^. Each field intersected 2 to 11 map unit polygons, with each map unit including several components. Soil type and properties for the map units’ major components were extracted at each yield map pixel. Then, soil profile data including texture and organic carbon was integrated to 1 m depth for analysis.

### Tests of hypotheses

We calculated variance components with hierarchical linear models with all random factors, to quantify the share of the variance in standardized yield (*sY*) that is accounted by four explanatory variables:

2 $$sY=\mu +{u}_{F}+{u}_{ST(F)}+{u}_{LP(ST(F))}+{u}_{WY(LP(ST(F)))}+\varepsilon $$

where *μ* is the overall mean, *u*_*F*_ is the random effect of field, *u*_*ST*(*F*)_ is the random effect of soil type (i.e., SSURGO map unit) within each field, *u*_*LP*(*ST*(*F*))_ is the random effect of landscape position within soil type-field combination, *u*_*WY*(*LP*(*ST*(*F*)))_ is the random effect of weather year within landscape position-soil type-field combination, and *ε* is the residual error. The analysis was then repeated by also including yield history as explanatory variable (*YH*).

To test the hypothesis that yield stability differs among landscape positions, we fitted the following linear mixed effects model to the binomial response of yield stability classification (*YSC*; i.e., stable or unstable):

3 $$\log \,it(YSC)=\mu +{\alpha }_{LP}+{u}_{S}+{u}_{S(F)}+{u}_{ST(S(F))}+\varepsilon $$

where *α*_*LP*_ are parameters depending on a pixel’s position in the landscape (summit, shoulder, midslope, toeslope, depression and flat), *u*_*S*_ is the random effect of state, *u*_*F*(*S*)_ is the random effect of field within state, and *u*_*ST*(*F*(*S*))_ is the random effect of soil type within field and state. We applied the logit function to generalize the binomial response to a linear model, and back transformed the results using the inverse logit function.

We also tested whether standardized yields in unstable pixels across landscape positions responded differently to May cumulative rainfall (MCR; mm), July cumulative rainfall (JCR; mm), and July mean daily maximum temperature (JMT, °C), using the following model:

4 $$sY=\mu +{\alpha }_{LP}\ast ({\beta }_{1}\ast MCR+{\beta }_{2}\ast JCR+{\beta }_{3}\ast JMT)+{u}_{F}+{u}_{ST(F)}+\varepsilon $$

where *β*_1_, *β*_2_ and *β*_3_ are regression slope coefficients. Finally, we tested differences in the standardized canopy temperatures (*sCT*) in unstable pixels among landscape positions:

5 $$sCT=\mu +{\alpha }_{LP}+{u}_{F}+\varepsilon $$

Differences among the six levels of landscape position were tested using the Tukey adjustment to correct for multiple comparison. In all tests, we rejected the null hypothesis (i.e., no effect) if the estimated probability was lower than 0.05. All test used the corn and soybean crop years only.

Given similar responses to weather (Fig. 2c), we pooled landscape positions into three classes: upland (summit, shoulder and midslope), lowland (toeslope and depression) and flat. We considered to be areas prone water deficit as those that exhibited unstable yields and were located in the upland position, whereas excess water-prone areas as those lowland positions with unstable yields. Then for every field and year, we computed their mean yield relative to the field-year mean yield.

To estimate yield penalty as a function of seasonal rainfall, we fitted OLS regression to the relative yield in areas prone to excess and deficit water, independently for each zone type and crop, with seasonal rainfall anomaly as the independent variable. This was computed by subtracting the site’s long-term (1988–2017) mean rainfall and scaling by the standard deviation.

We modeled the response of field mean yield ($\bar{y}$) to seasonal (May—Sep) rainfall using Rickter’s curve^43^:

6 $$\bar{y}=a\ast (x-c)\ast {\exp }(-b\ast (x-c))$$

where *x* is independent variable of seasonal rainfall anomaly, and *a*, *b*, and *c*, are the fitted constants. The models were computed independently for corn and soybean. Finally, we linked the OLS regressions with the non-linear model, so that the idealized response of yield (*y*′) in drought- or flood prone areas was:

7 $$y{\prime} =({\beta }_{0}+{\beta }_{1}\ast x)\ast \bar{y}$$

where *β*_0_, and *β*_1_ are the OLS regression parameters.

### Up-scaling to regional-level impacts

To assess regional-level yield gaps in drought and flood-prone subfield zones and associated impacts, we use a satellite-derived yield stability classification of corn and soybean cropland for 10 Midwestern states^13^ (Fig. S12). This yield stability classification is based on temporal variability in NDVI derived from Landsat 5, 7, and 8 images (30 m resolution) collected between 2010 and 2017. Details about the image classification procedure, as well as ground-truth validation, are provided by a previous study^13^. Yield stability classifications were laid over regional NED-DEMs to determine the share of unstable yield zones in upland, lowland or flat areas. Total cropland area and the percentage of cropland in unstable lowland and upland was aggregated for each county. We excluded urban counties (population > 100,000) and those with <1,000 ha under corn or soybean cultivation.

We estimated county-level yields in water-excess and water-deficit prone cropland (*y*′) for the period 2007 to 2016 using Eq. (7). In this case, $\,\bar{y}$ is the average county yield as reported by the National Agricultural Statistic Service^44^ (NASS; Figs. S13 and S14), and *x* is the seasonal (May-Sep) rainfall anomaly for each US climate division (Fig. S15). Monthly rainfall values for each climate division are averages computed using daily observations from individual weather stations. Climate data was accessed through the Iowa Environmental Mesonet Climodat web portal^45^.

### Software

All data processing and analysis was conducted in R (version 3.5.2) extended with the following packages: *daymetr* to query Daymet data; *FedData* to access NED and SSURGO databases; *sp* and *raster* to process spatial data and terrain analysis; *dplyr* and *tidyr* for all other data wrangling; *lme4* for linear-mixed effects model fitting*; emmeans* for post-hoc mean comparison; *nlme* for non-linear model fitting; and *rasterVis* and *ggplot2* for data visualization.

## Supplementary information

Supplementary Information.
