# Supplementary material for: Unstable crop yields reveal opportunities for site-specific adaptations to climate variability
Source: Sci Rep. 2020 Feb 19;10:2885. doi: 10.1038/s41598-020-59494-2 (PMC7031360; doi:10.1038/s41598-020-59494-2)

Unstable crop yields reveal opportunities for site-specific adaptations to climate change

Rafael A. Martinez-Feria and Bruno Basso*

Department of Earth and Environmental Sciences, Michigan State University, East Lansing, MI, USA

* Corresponding author: Bruno Basso (basso@msu.edu)

SUPPLEMENTAL INFORMATION

Supplemental Tables 2

Supplemental Table S1. Description summary of the sample fields used in the analysis 2

Supplemental Table S2. Test of hypothesis for the effect of landscape position on yield stability. 3

Supplemental Table S3. Test of hypothesis for the effect of landscape position on the standardized crop yield response to weather covariates. 4

Supplemental Table S4. Test of hypothesis for the effect of landscape position on the standardized crop canopy temperature in July. 5

Supplemental Figures 6

Supplemental Figure S1. Share of cropland in sample fields under six landscape position classes 6

Supplemental Figure S2. Share of variance in standardized yield explained by grouping factors at individual sample fields..... 7

Supplemental Figure S3. Yield stability by region and landscape position positions. 8

Supplemental Figure S4. Standardized yield response to weather across landscape positions by region. 9

Supplemental Figure S5. Standardized yield response to weather across landscape positions by crop. 10

Supplemental Figure S6. Canopy temperature sensing. 11

Supplemental Figure S7. Differences in soil organic carbon across landscape positions. 12

Supplemental Figure S8. Differences in soil texture across landscape positions. 13

Supplemental Figure S9. Differences in soil depth across landscape positions. 14

Supplemental Figure S10. Relative crop yield response to seasonal rainfall anomaly across landscape positions. 15

Supplemental Figure S11. Response of the field average yield to seasonal rainfall anomaly. 16

Supplemental Figure S12. Satellite-derived crop yield stability classes. 17

Supplemental Figure S13. NASS -reported county-level average corn yields for 2007-2016 18

Supplemental Figure S14. NASS-reported county-level average soybean yields for 2007-2016 19

Supplemental Figure S15. Seasonal rainfall anomaly for US Climate division in the study area during 2007-2016 20

SUPPLEMENTAL TABLES

Supplemental Table S1. Description summary of the sample fields used in the analysis

Ecoregion (Level III)*	State	Abbreviation	Farmers	Fields	Crops**	Area (ha)		Site-years	
						Total	*Average*	*Total*	*Average*
Western Corn-belt plains (47)	Minnesota	MN	6	9	Corn, Soybean	451	50.1	27	3
	Iowa	IA	32	62	Corn, Soybean	2,993	48.3	408	6.6
Central Corn-belt plains (54)	Illinois	IL	9	13	Corn, Soybean	582	44.8	40	3.1
	Indiana	IN	1	2	Corn, Soybean	71	35.5	21	10.5
Drift plains (56)	Michigan	MI	17	220	Corn, Soybean, Wheat	6,447	29.3	1209	5.5
**All**			**65**	**306**		**10,544**	**34.6**	**1705**	**5.7**

* As defined by U.S. Environmental Protection Agency - National Health and Environmental Effects Laboratory (https://www.epa.gov/eco-research/level-iii-and-iv-ecoregions-continental-united-states)

**In order of frequency

Supplemental Table S2. Test of hypothesis for the effect of landscape position on yield stability.

	Estimate	Std. error	P value
Fixed effects			
μ	-1.126	0.337	0.0008
LP_{Summit}	0.332	0.029	<0.0001
$LP_{Shoulder}$	0.184	0.282	<0.0001
$LP_{Midslope}$	0.205	0.288	<0.0001
$LP_{Toeslope}$	0.245	0.027	<0.0001
$LP_{Depression}$	0.553	0.029	<0.0001
Random effects	σ^2 explained		
ST(F(S))	0.94		
F(S)	0.72		
S	0.55		
ε	1.00		

μ = intercept; LP= landscape position within soil type; ST(F(S)) = soil type within field and state; F(S) = field within state; S = state; ε = Residual error.

Supplemental Table S3. Test of hypothesis for the effect of landscape position on the standardized crop yield response to weather covariates.

	Both			Corn			Soybean		
	Estimate	Std. error	P-value	Estimate	Std. error	P-value	Estimate	Std. error	P-value
Fixed effects									
μ	-0.599	0.102	<0.001	-0.362	0.130	0.005	-0.820	0.173	<0.001
LP_{Summit}	0.424	0.145	0.003	0.019	0.181	0.915	0.642	0.261	0.014
$LP_{Shoulder}$	0.089	0.167	0.595	-0.377	0.207	0.068	0.536	0.287	0.061
$LP_{Midslope}$	0.113	0.153	0.459	-0.155	0.189	0.411	-0.125	0.279	0.655
$LP_{Toeslope}$	-0.554	0.150	<0.001	-0.683	0.189	<0.001	-0.552	0.250	0.027
$LP_{Depression}$	-0.779	0.139	<0.001	-0.769	0.175	<0.001	-1.072	0.245	<0.001
JCR	0.000	0.000	0.182	0.000	0.000	0.045	0.000	0.000	0.537
MCR	0.000	0.000	0.011	-0.001	0.000	<0.001	0.000	0.000	0.407
JMT	0.004	0.003	0.264	-0.002	0.004	0.573	0.017	0.006	0.006
LP_{Summit}: JCR	0.001	0.000	<0.001	0.002	0.000	<0.001	0.001	0.000	0.003
$LP_{Shoulder}$: JCR	0.001	0.000	<0.001	0.001	0.000	0.028	0.002	0.000	0.000
$LP_{Midslope}$: JCR	-0.001	0.000	<0.001	-0.001	0.000	0.003	0.000	0.001	0.784
$LP_{Toeslope}$: JCR	-0.001	0.000	<0.001	-0.001	0.000	0.042	-0.003	0.000	<0.001
$LP_{Depression}$: JCR	-0.002	0.000	<0.001	-0.001	0.000	0.021	-0.004	0.000	<0.001
LP_{Summit}: MCR	0.000	0.000	0.086	0.001	0.000	0.000	0.000	0.000	0.595
$LP_{Shoulder}$: MCR	0.001	0.000	0.005	0.001	0.000	0.003	0.001	0.000	0.252
$LP_{Midslope}$: MCR	0.001	0.000	0.020	0.001	0.000	<0.001	0.000	0.000	0.565
$LP_{Toeslope}$: MCR	-0.001	0.000	<0.001	-0.001	0.000	0.015	-0.002	0.000	<0.001
$LP_{Depression}$: MCR	-0.002	0.000	<0.001	-0.002	0.000	0.000	-0.002	0.001	<0.001
LP_{Summit}: JMT	-0.029	0.005	<0.001	-0.019	0.006	0.001	-0.034	0.009	<0.001
$LP_{Shoulder}$: JMT	-0.015	0.006	0.009	0.002	0.007	0.800	-0.034	0.010	0.001
$LP_{Midslope}$: JMT	-0.008	0.005	0.146	-0.001	0.006	0.814	0.002	0.010	0.855
$LP_{Toeslope}$: JMT	0.030	0.005	<0.001	0.028	0.006	<0.001	0.039	0.009	<0.001
$LP_{Depression}$: JMT	0.042	0.005	<0.001	0.039	0.006	<0.001	0.061	0.009	<0.001
Random effects				σ^2 explained					
ST(F)	0.54			0.59			0.64		
F	0.37			0.36			0.29		
ε	1.26			1.23			1.26		

μ = intercept; MCR = May cumulative rainfall; MCR = July cumulative rainfall; JMT = July mean daily maximum temperature; LP= landscape position within soil type; ST(F) = soil type within field; F = field; ε = Residual error.

Supplemental Table S4. Test of hypothesis for the effect of landscape position on the standardized crop canopy temperature in July.

	Estimate	Std. error	P value
Fixed effects			
μ	-0.030	0.006	<0.001
LP_{Summit}	0.132	0.010	<0.001
$LP_{Shoulder}$	0.067	0.011	<0.001
$LP_{Midslope}$	0.053	0.011	<0.001
$LP_{Toeslope}$	-0.034	0.011	0.002
$LP_{Depression}$	-0.003	0.011	0.757
Random effects	σ^2 explained		
F	0.01		
ε	0.56		

μ = intercept; F = field; ε = Residual error.

SUPPLEMENTAL FIGURES

Supplemental Figure S1. Share of cropland in sample fields under six landscape position classes

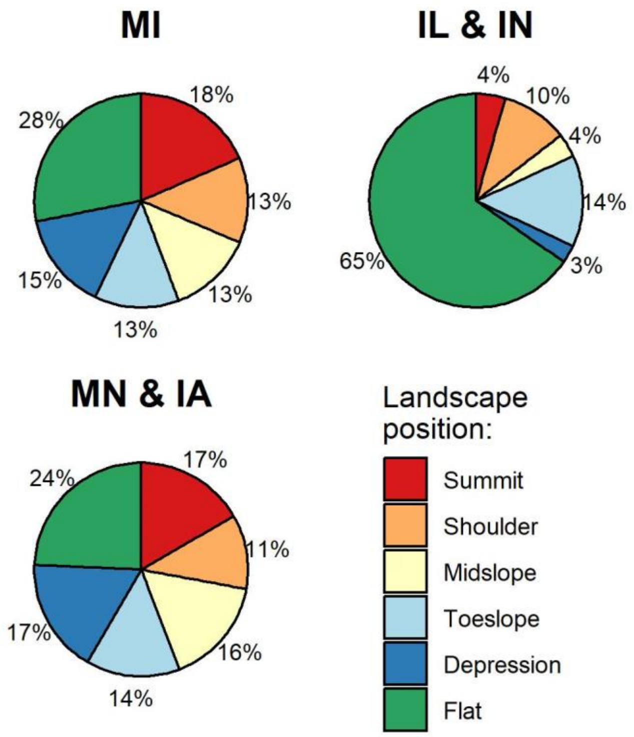

Total subfield area reported for each field within each distinct ecoregion (Table S1). MN = Minnesota, IA = Iowa, IL = Illinois, IN = Indiana, MI = Michigan.

Supplemental Figure S2. Share of variance in standardized yield explained by grouping factors at individual sample fields.

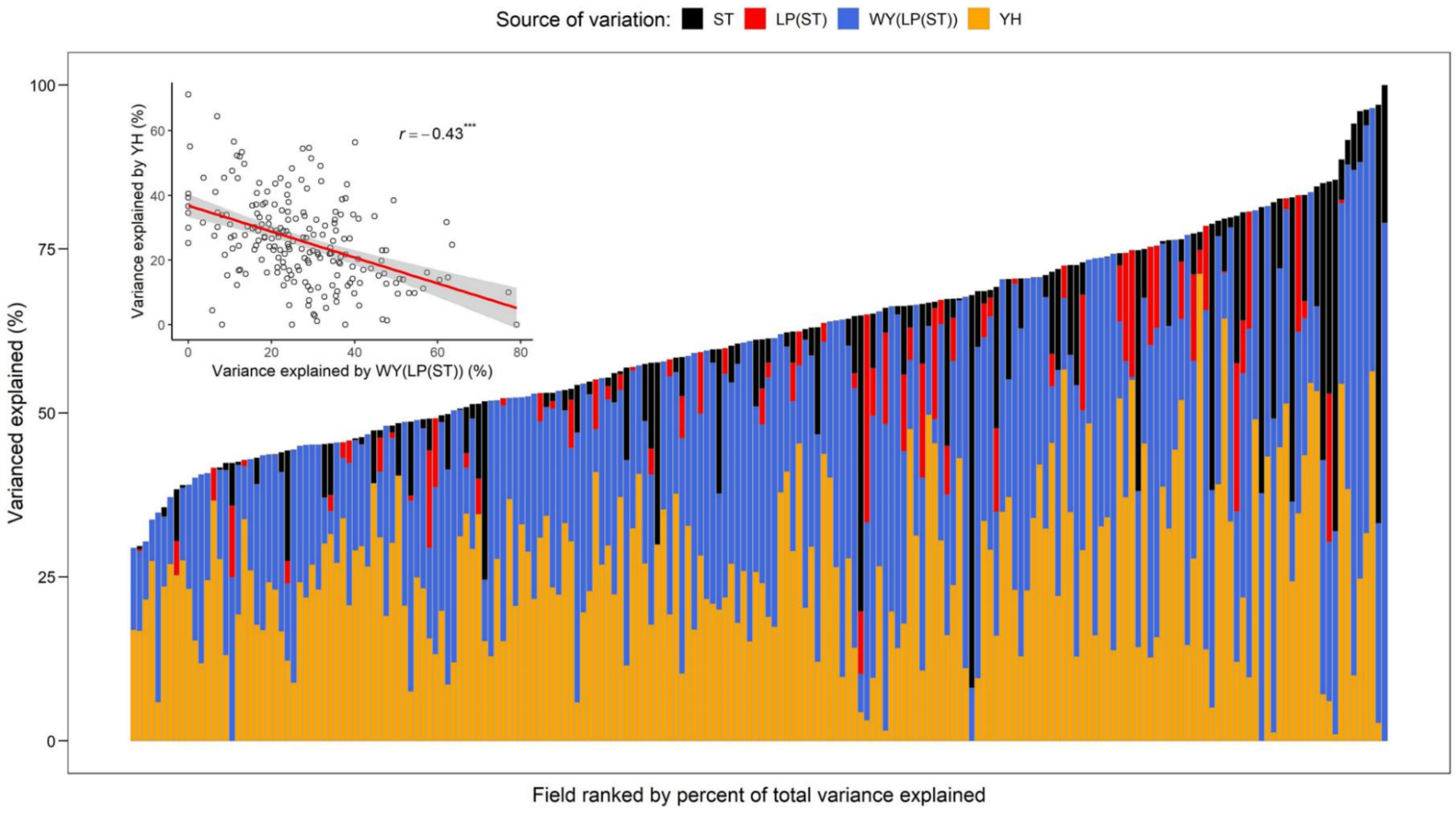

ST = soil type; LP(ST) = landscape position within soil type. WY(LP(ST)) = weather year within landscape position and soil type; YH = yield history (i.e., whether average standardized yield is above or below zero).

Supplemental Figure S3. Yield stability by region and landscape position positions.

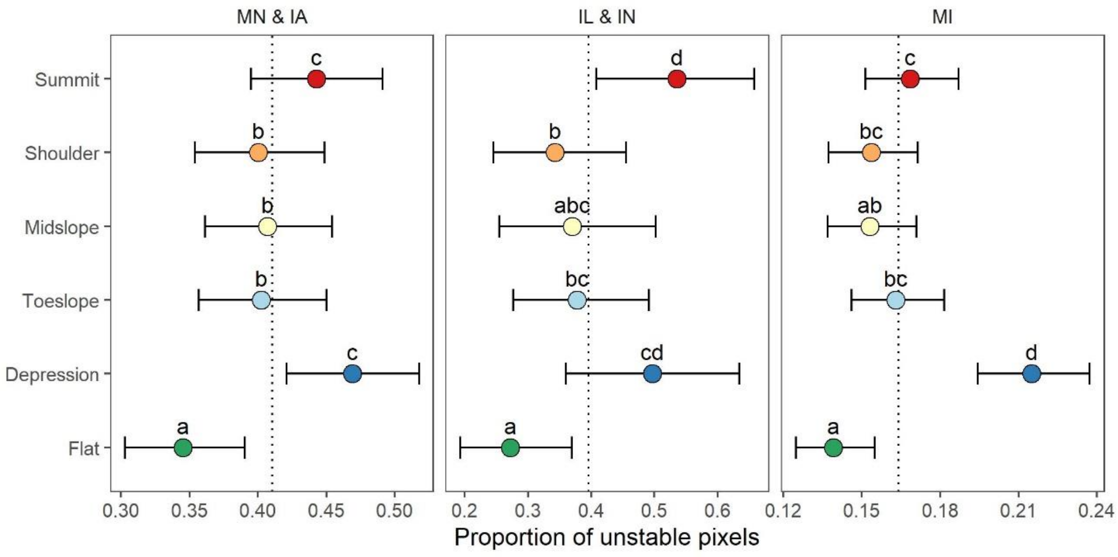

Proportion of unstable yield map pixels, that is with a large degree of temporal variability. MN = Minnesota, IA = Iowa, IL = Illinois, IN = Indiana, MI = Michigan. Landscape positions with the same letter indicate that they are not significantly different (Tukey adjustment, $p < 0.05$).

Supplemental Figure S4. Standardized yield response to weather across landscape positions by region.

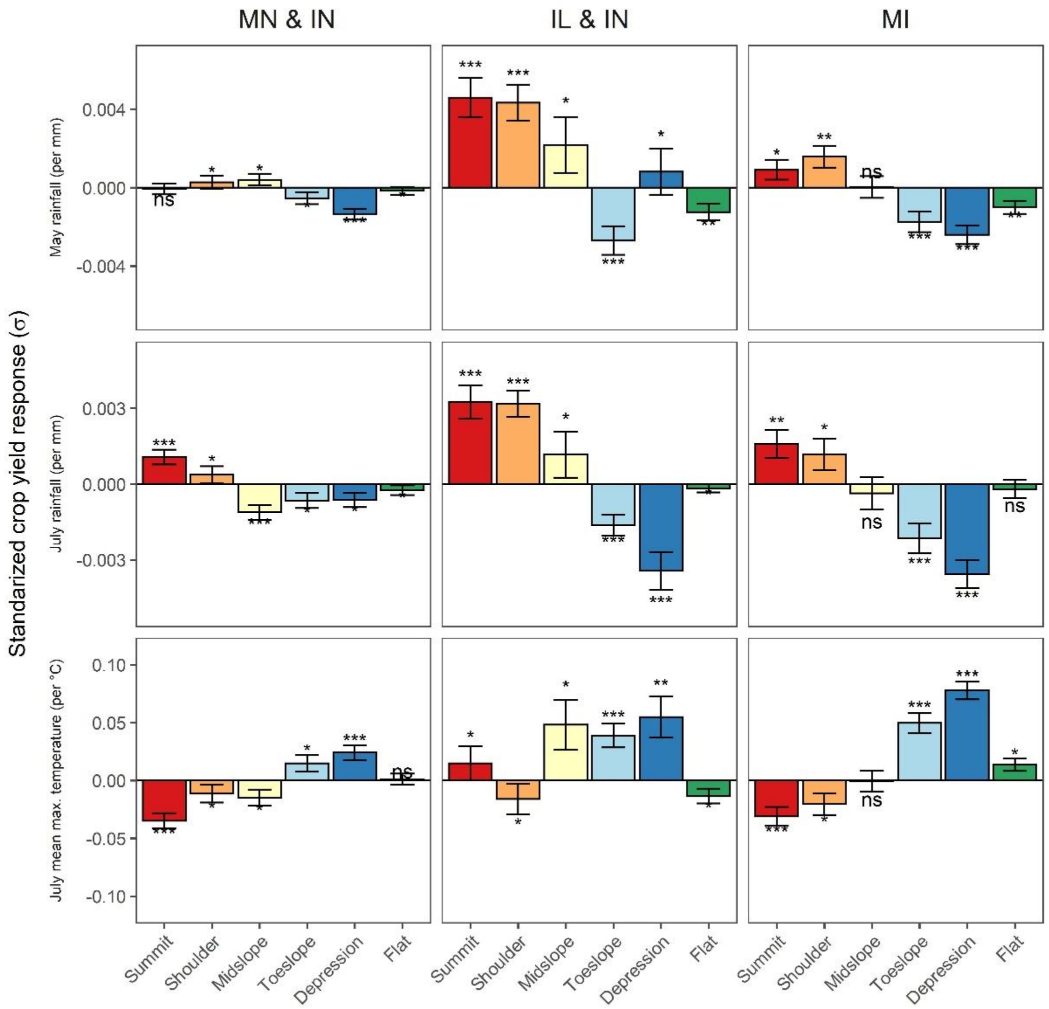

Significance codes: ns = non-significant, * = p < 0.05, ** = p < 0.01; *** = p < 0.001. Error bars indicate standard error of the estimate. MN = Minnesota, IA = Iowa, IL = Illinois, IN = Indiana, MI = Michigan.

Supplemental Figure S5. Standardized yield response to weather across landscape positions by crop.

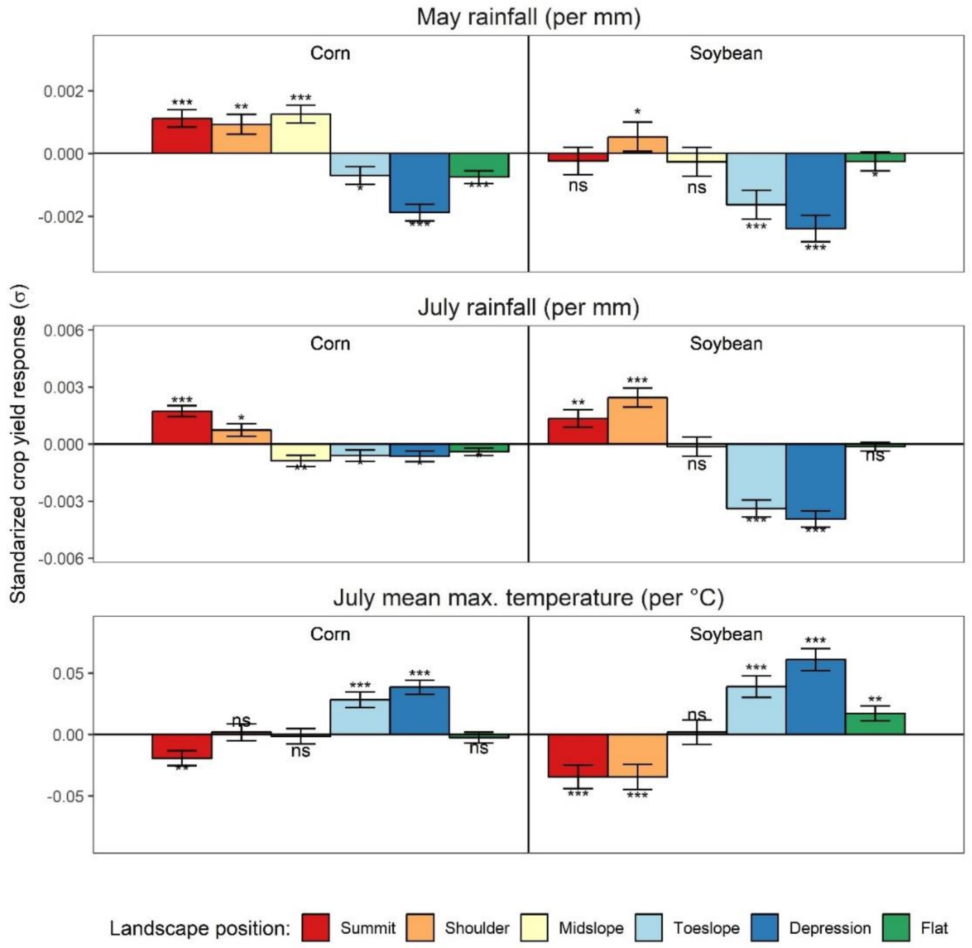

Significance codes: ns = non-significant, * = p < 0.05, ** = p < 0.01; *** = p < 0.001. Error bars indicate standard error of the estimate

Supplemental Figure S6. Canopy temperature sensing.

(a)

(b)

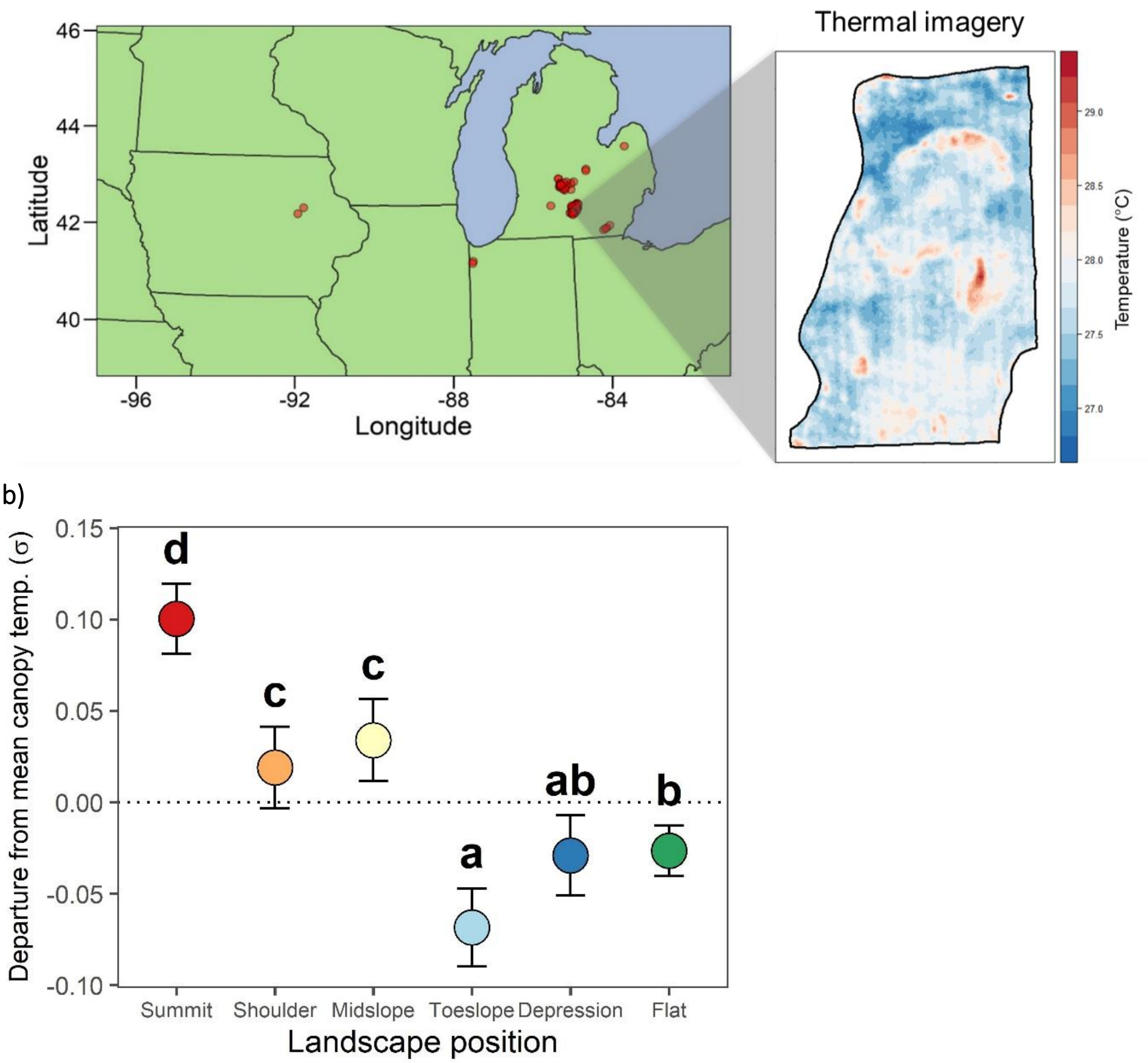

(a) Thermal imagery was collected in a subset of 60 fields during the month of July within 2013 and 2016, using an aircraft-mounted sensor (thermal band: 7-14 µm) at a 2 m resolution. **(b)** Differences in standardized canopy temperature readings collected in July and Agust across landscape positions. Landscape positions with the same letter indicate that they are not significantly different (Tukey adjustment, $p < 0.05$).

Supplemental Figure S7. Differences in soil organic carbon across landscape positions.

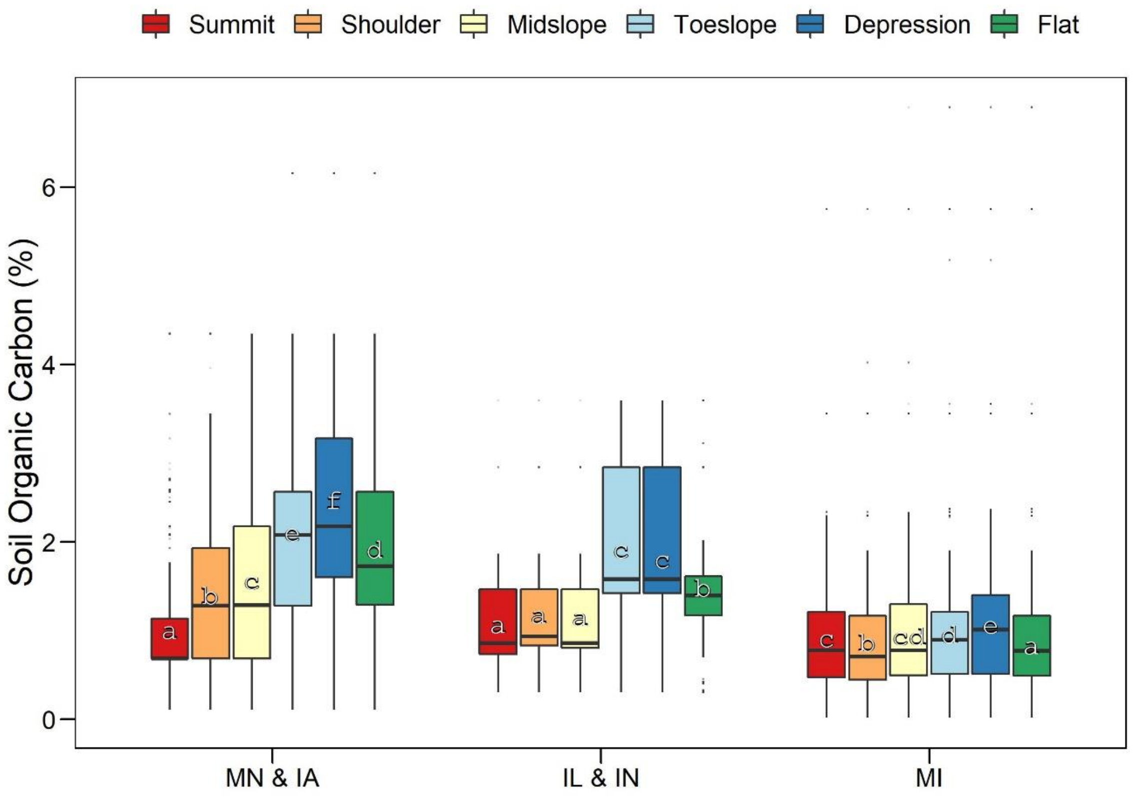

Derived from values extracted from SSURGO map unit polygons (see Methods in main text). Landscape positions with the same letter indicate not significantly different (Tukey adjustment, $p < 0.05$). MN = Minnesota, IA = Iowa, IL = Illinois, IN = Indiana, MI = Michigan.

Supplemental Figure S8. Differences in soil texture across landscape positions.

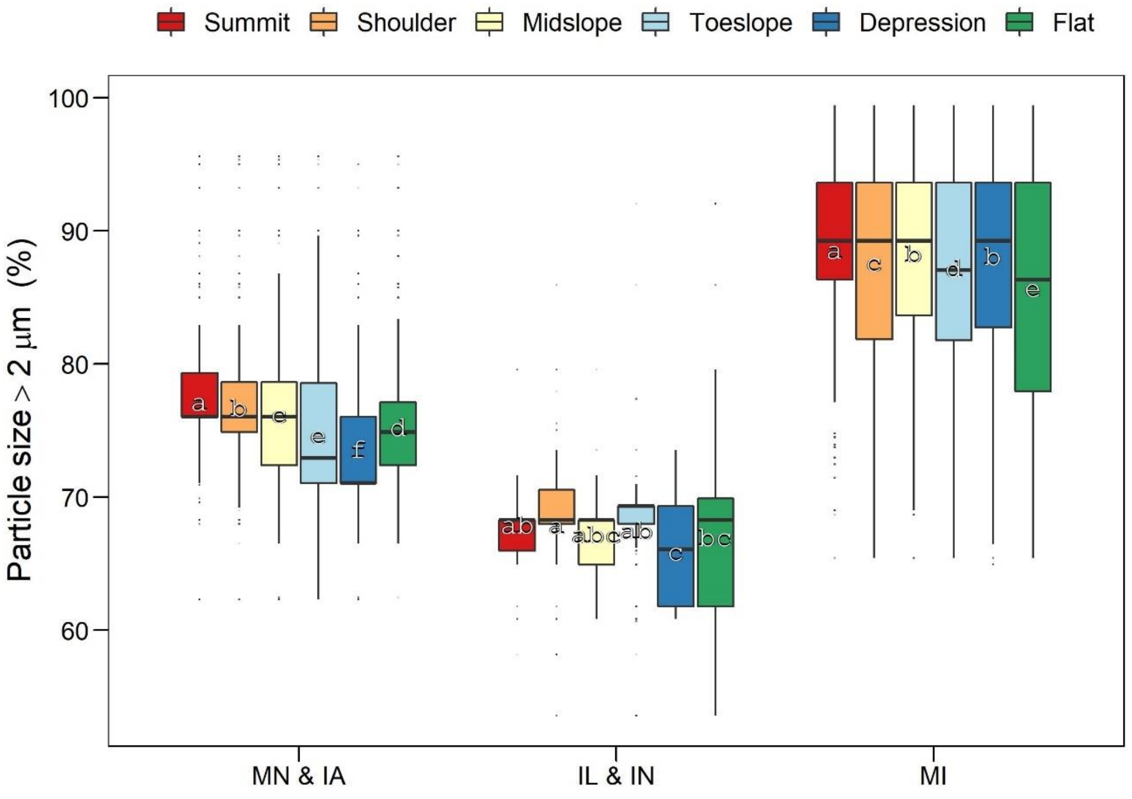

Derived from values extracted from SSURGO map unit polygons (see Methods in main text). Landscape positions with the same letter indicate not significantly different (Tukey adjustment, $p < 0.05$). MN = Minnesota, IA = Iowa, IL = Illinois, IN = Indiana, MI = Michigan.

Supplemental Figure S9. Differences in soil depth across landscape positions.

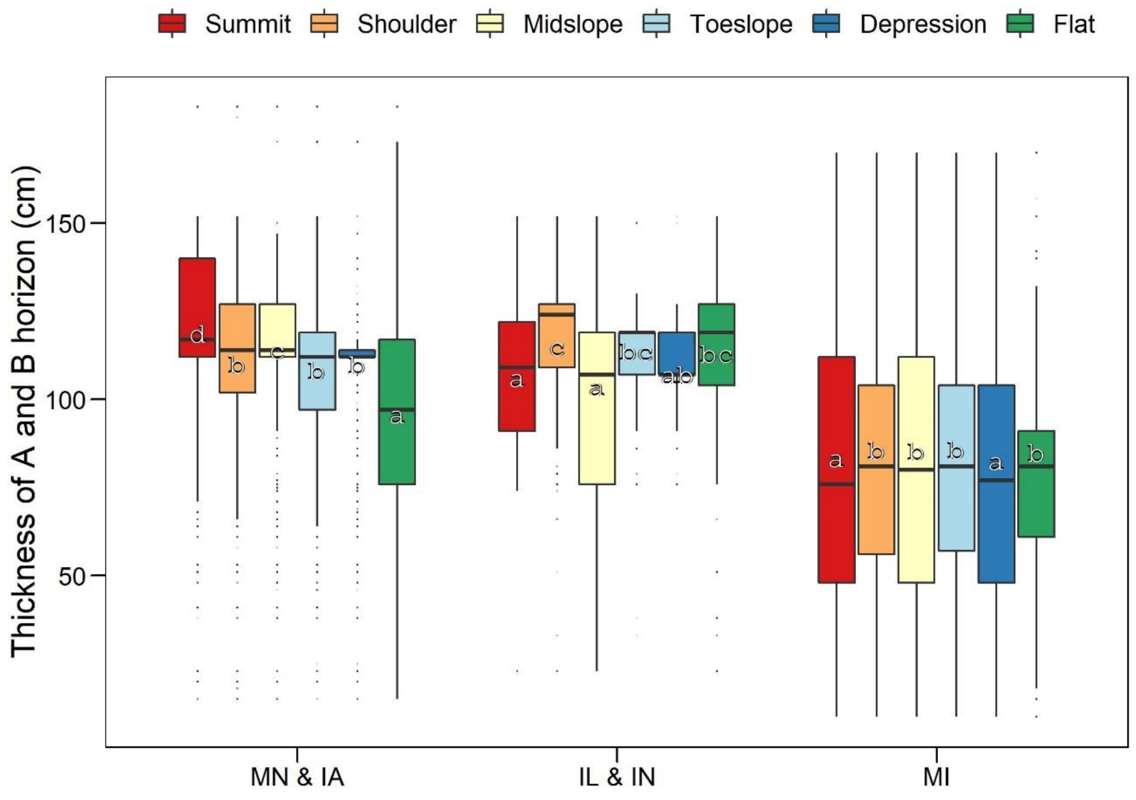

Derived from values extracted from SSURGO map unit polygons (see Methods in main text). Landscape positions with the same letter indicate not significantly different (Tukey adjustment, $p < 0.05$). MN = Minnesota, IA = Iowa, IL = Illinois, IN = Indiana, MI = Michigan.

Supplemental Figure S10. Relative crop yield response to seasonal rainfall anomaly across landscape positions.

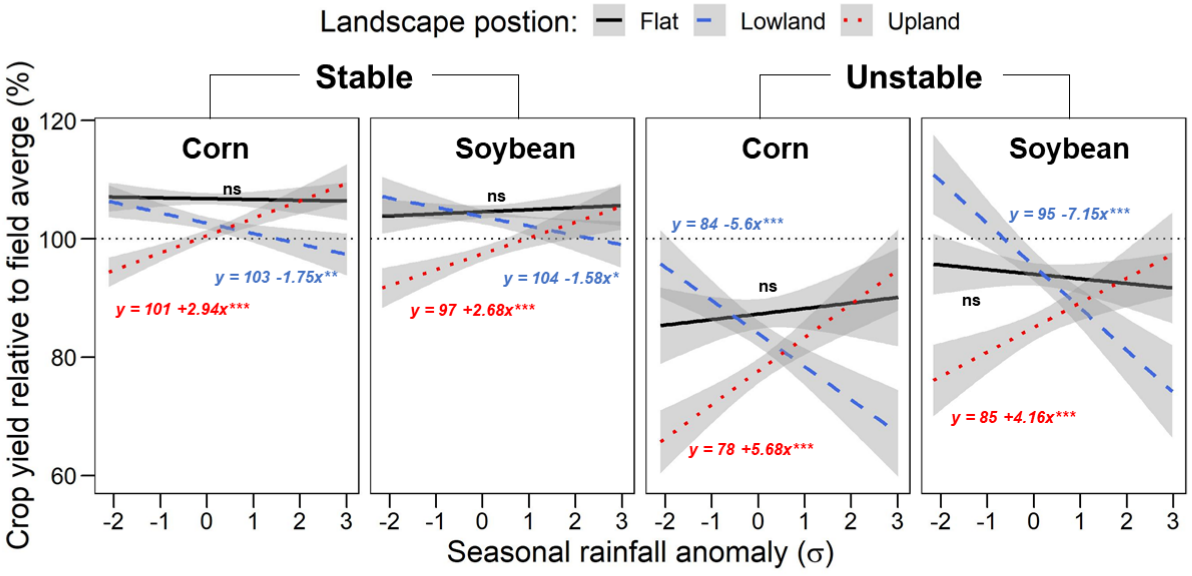

Ordinary linear regressions (OLS) of the mean yield of each zone classification relative to the site-year average yield of the field in response to cumulative seasonal (May to September) rainfall anomaly. The latter is calculated as the deviation from the long-term mean rainfall (1987-2016) scaled by the standard deviation. Significance codes: ns = non-significant, * = $p < 0.05$, ** = $p < 0.01$; *** = $p < 0.001$. Ribbon around the regression line indicates standard error of the fit.

Supplemental Figure S11. Response of the field average yield to seasonal rainfall anomaly.

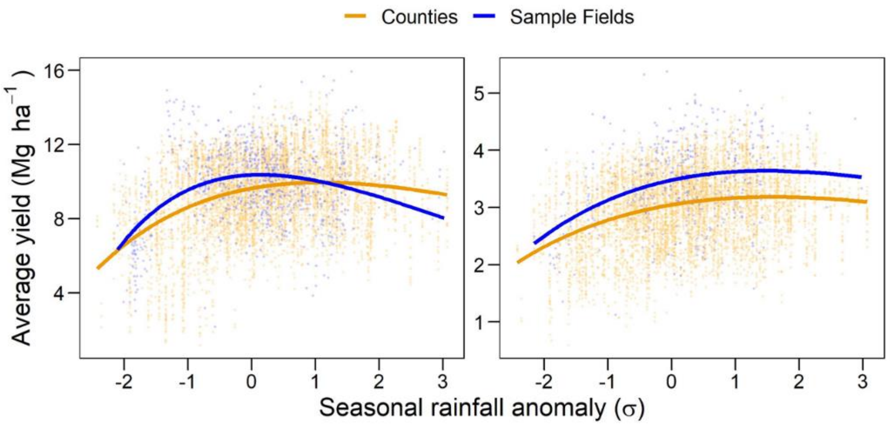

$$y = a * (x - c) * \exp(-b * (x - c))$$

		Parameter		
		a	***b***	***c***
**Sample Fields**				
	Corn	8.7 (0.53)	0.31 (0.018)	3.1 (0.12)
	Soybean	1.8 (0.32)	0.18 (0.034)	4 (0.49)
**Counties**				
	Corn	5.7 (0.19)	0.21 (0.0072)	3.6 (0.089)
	Soybean	1.4 (0.069)	0.17 (0.0084)	4.4 (0.16)

All non-linear regression parameter estimates (standard errors in parenthesis) were significant at the $p < 0.05$ level. Cumulative seasonal (May to September) rainfall anomaly is calculated as the deviation from the long-term average rainfall (1987-2016) scaled by the standard deviation. County-average yields are as reported by NASS during 2007-2016 (see Methods in main text and Figs. S11-12).

Supplemental Figure S12. Satellite-derived crop yield stability classes.

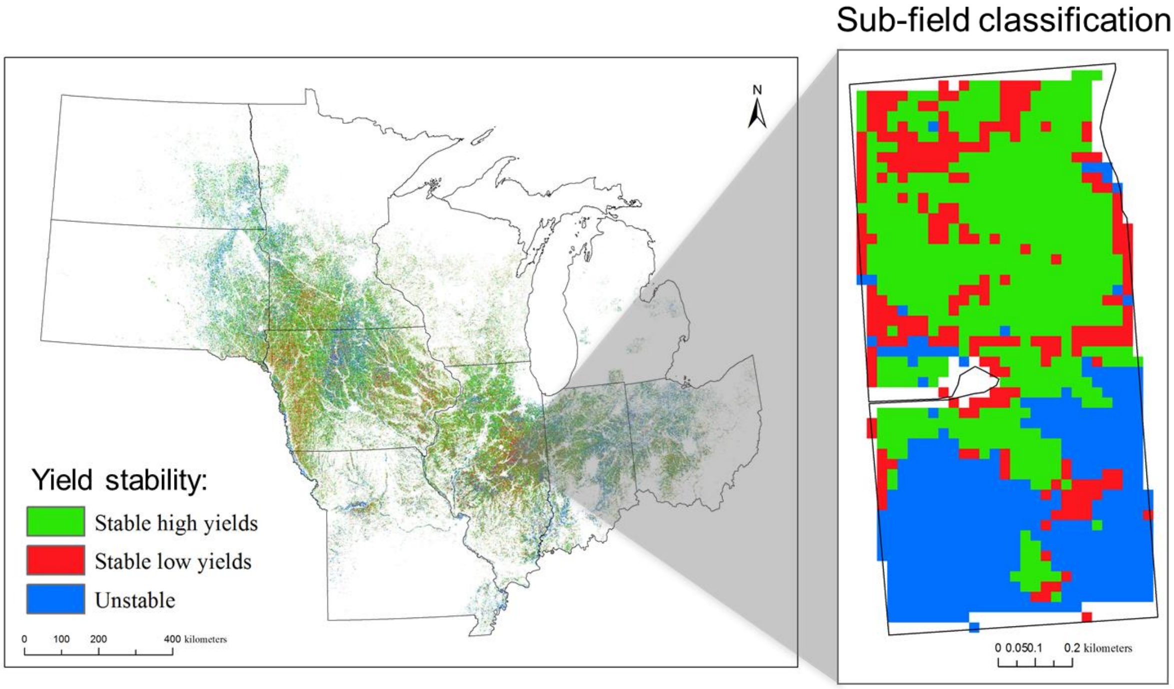

Subfield crop yield stability classes dataset is based on the temporal variability in NDVI (normalized difference vegetation index) from Landsat 5, 7, and 8 images (30 m resolution), collected between 2010 and 2017 (see Basso et al., *Sci. Rep*. **9**, 5774 (2019) for details). Cropland map is partitioned in stable-high (green), stable-low (red) and unstable (blue) zones.

Supplemental Figure S13. NASS -reported county-level average corn yields for 2007-2016

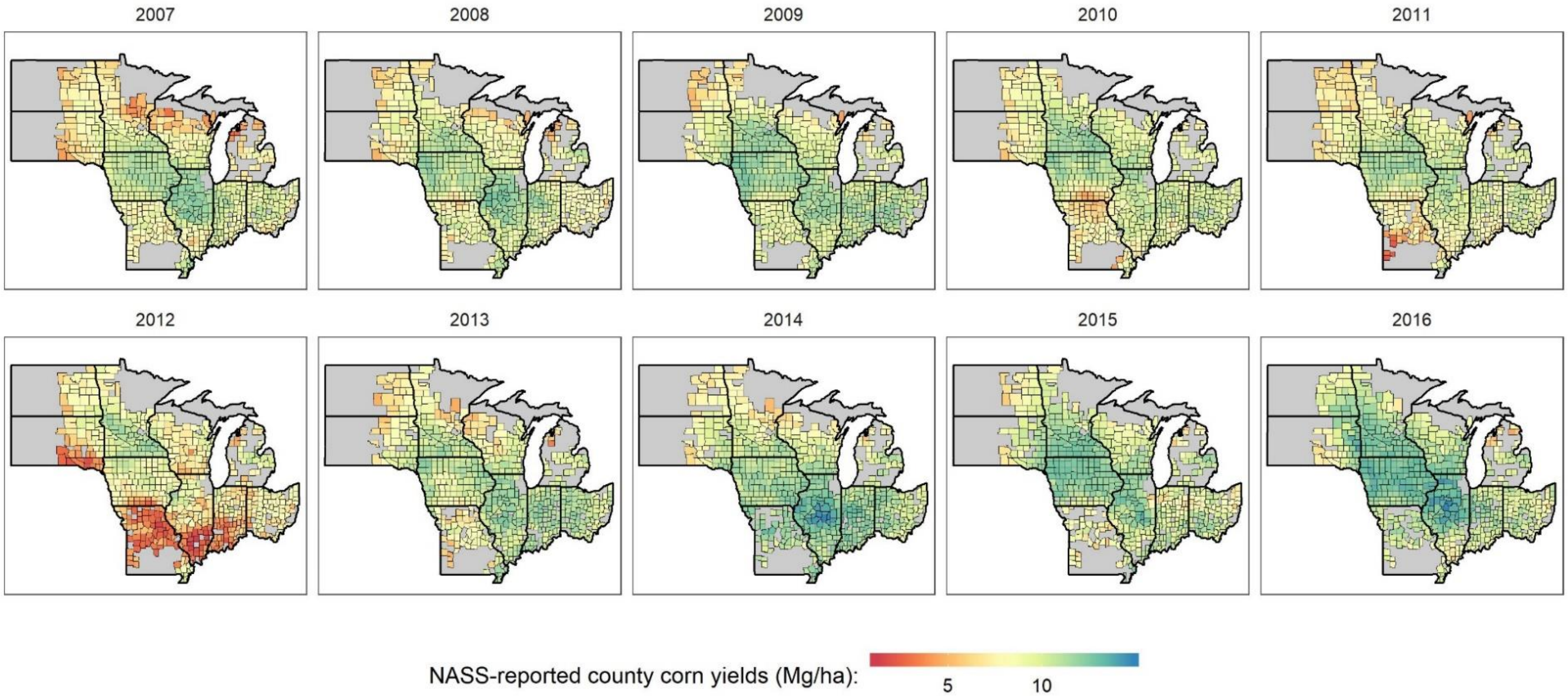

Supplemental Figure S14. NASS-reported county-level average soybean yields for 2007-2016

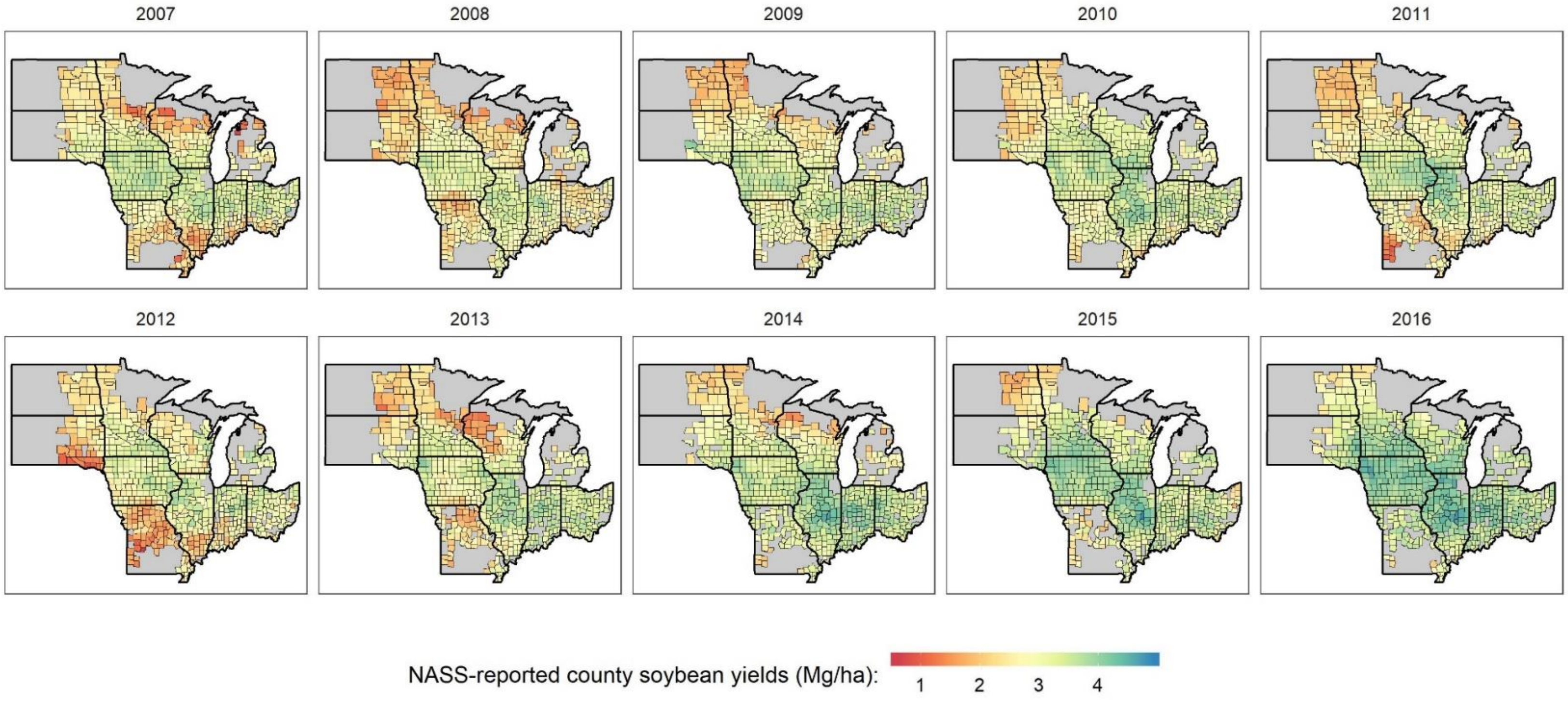

Supplemental Figure S15. Seasonal rainfall anomaly for US Climate division in the study area during 2007-2016

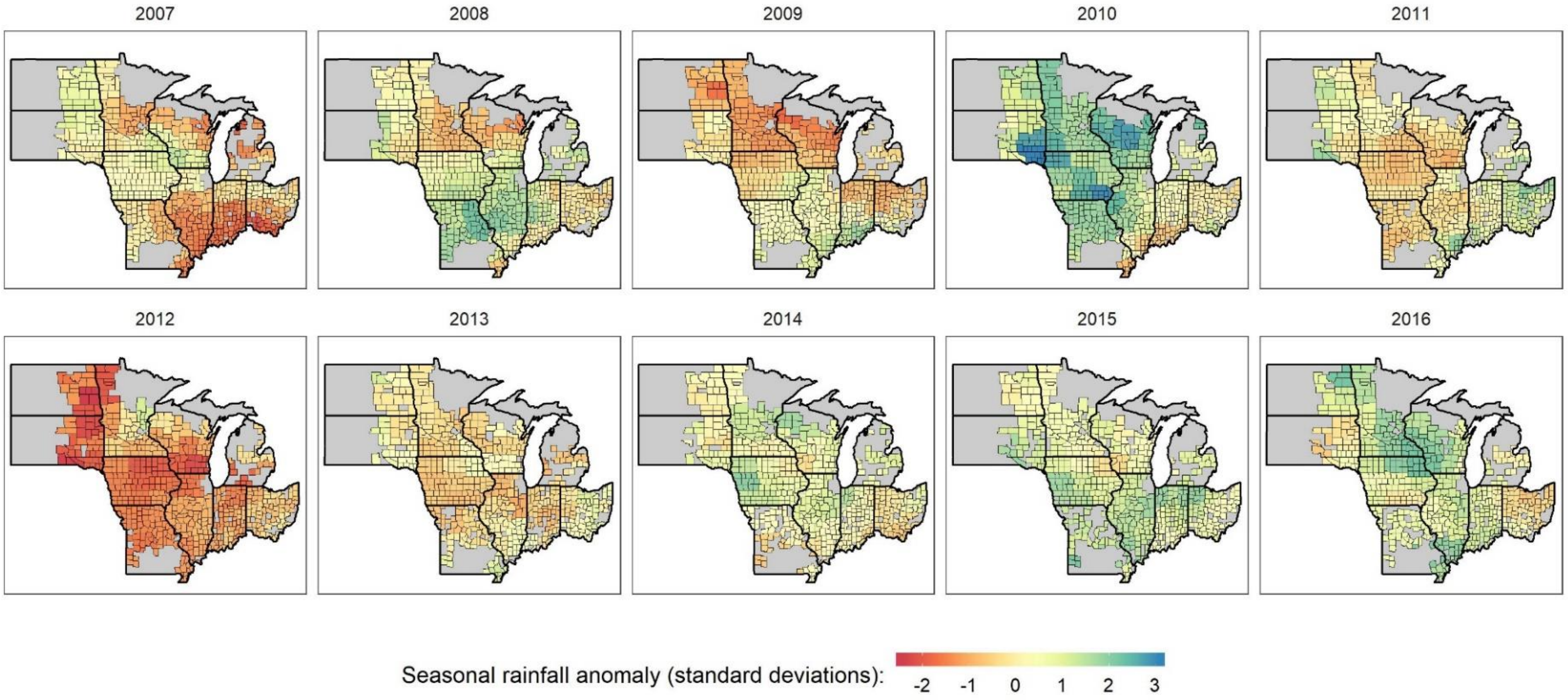

Supplement: Supplementary file 1 — Supplementary Information. [file 41598_2020_59494_MOESM1_ESM.pdf]
